# Dryland Soil Rewetting Induces Strong Volatile Organic Compound Emissions With Potential to Form Ozone and Aerosols

**DOI:** 10.1111/gcb.71145

**Published:** 2026-10-07

**Authors:** Andrea Ghirardo, Benjamin W. Poodiack, Hagar Siebner, Matan K. Jaffe, Baris Weber, Jörg‐Peter Schnitzler, Michael Bonkowski, Osnat Gillor, Alex B. Guenther

**Affiliations:** ^1^ Research Unit Environmental Simulation Helmholtz Center Munich Neuherberg Germany; ^2^ Institute for Clinical Nutritional Medicine Technical University of Munich, TUM School of Medicine and Health, TUM University Hospital, TUM Campus at the Olympiapark Munich Germany; ^3^ Else Kröner‐Fresenius‐Center for Nutritional Medicine Technical University of Munich Freising Germany; ^4^ Institute for Translational Metabolism Research Helmholtz Diabetes Center at Helmholtz Munich Neuherberg Germany; ^5^ German Center for Diabetes Research (DZD) Neuherberg Germany; ^6^ Zuckerberg Institute for Water Research, Blaustein Institutes for Desert Research Ben‐Gurion University of the Negev Midreshet Ben Gurion Israel; ^7^ Department of Software and Information Systems Engineering, Faculty of Computer and Information Science Ben Gurion University of the Negev Be'er‐Sheva Israel; ^8^ Institute of Zoology, Terrestrial Ecology, Cluster of Excellence on Plant Sciences (CEPLAS) University of Cologne Cologne Germany; ^9^ Department of Earth System Science University of California Irvine Irvine California USA

## Abstract

Drylands cover nearly half of Earth's terrestrial surface and are expanding under climate change. Biogenic volatile organic compounds (VOCs) significantly influence atmospheric chemistry, yet microbial VOC emissions from dryland soils remain poorly understood. Here, we investigate VOC emissions associated with petrichor—the characteristic scent following rainfall after prolonged drought—across a steep aridity gradient extending into the Negev Desert. Soil rewetting triggered strong VOC fluxes (1–3.5 nmol m^−2^ s^−1^ ground area) dominated by sesquiterpenes and benzenoids, with emission patterns and rates linked to climatic regions, soil aridity, and microbial community composition. Petrichor consisted of a complex bouquet of 58 VOCs, and emission fluxes showed a pronounced initial burst. We upscaled the results to Israel and show that petrichor emissions contribute significantly to ozone and secondary organic aerosol formation potentials, comparable in magnitude to those estimated for anthropogenic VOC sources. Soil aridity shaped VOC chemical composition and emission dynamics, indicating strong microbial and environmental controls on the fluxes. As climate change alters precipitation regimes and promotes dryland expansion, these rainfall‐driven bursts of microbial VOCs may represent an overlooked climate‐microbe feedback influencing atmospheric chemistry and radiative forcing at regional and global scales.

## Graphical abstract

Rainfall after drought triggers strong petrichor VOC emissions from dryland soils. These emissions are chemically diverse, shaped by aridity and soil microbial community, and can increase regional ozone and secondary organic aerosol formation potentials. Dryland soil rewetting therefore represents an episodic but potentially important biosphere‐atmosphere feedback under climate change.

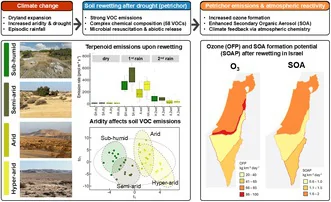

## 1 Introduction

Biogenic volatile organic compounds (VOCs) are reactive molecules that play a central role in atmospheric chemistry (Lelieveld et al. 2008; Machado et al. 2024). In the atmosphere, VOCs react with hydroxyl radicals (OH), contribute to ozone (O_3_) formation, and lead to the production of secondary organic aerosol (SOA), which can act as cloud condensation nuclei. These processes modulate atmospheric oxidative capacity and radiative forcing, thereby influencing air quality, cloud formation, and climate (Scott et al. 2018; Tripathi et al. 2025).

Globally, vegetation is the dominant source of terrestrial biogenic VOCs due to its large biomass and widespread, high terpenoid emissions (Lelieveld et al. 2008). Consequently, global VOC emission inventories [e.g., MEGAN (Sindelarova et al. 2014)] rely primarily on vegetation type and cover. However, current emission models generally omit soil VOC exchange, despite increasing evidence that soils can act both as sources and sinks of VOCs, thereby affecting net ecosystem‐atmosphere VOC exchange (Jiao et al. 2023; Kramshoj et al. 2018; Penuelas et al. 2014; Pugliese et al. 2026; Rinnan and Albers 2020). Soil wetting and rainfall events have been shown to trigger chemically diverse VOC emissions, including sesquiterpenes and other isoprenoids from Amazonian and rainforest soils, as well as oxygenated VOCs, sulfur‐containing compounds, and hydrocarbons from agricultural, grassland, and forest soils (Bourtsoukidis et al. 2018; Pugliese et al. 2023; Rossabi et al. 2018). These findings suggest that soils can contribute substantially to VOC fluxes, yet the magnitude and importance of these fluxes to atmospheric reactivity, especially relative to other ecosystems, remain largely unknown.

Drylands, which include arid, semi‐arid, and dry sub‐humid regions, cover nearly half of Earth's land surface (~66.7 Mkm^2^) (Imminger et al. 2024; Reynolds et al. 2007) and are expanding under climate change (Huang et al. 2017; Prăvălie 2016; Yao et al. 2020). Although dryland soils often support sparse vegetation, they host abundant, metabolically versatile microbial communities (Bay et al. 2020, 2021), making them an underexplored source of biogenic VOCs with unknown atmospheric relevance. Water availability strongly structures microbial community composition and function along aridity gradients (Bay et al. 2021). In hyper‐arid regions, microbes persist via dormancy or hygroscopic water uptake (Crits‐Christoph et al. 2018). In contrast, in less arid regions, episodic hydration events rapidly activate microbial metabolism (Delgado‐Baquerizo et al. 2017), and cooperative interactions further enhance survival in extremely dry soils (Maestre et al. 2012).

Climate change is expected to intensify precipitation variability and expand drylands globally, increasing the frequency of prolonged drought followed by episodic rainfall events (Huang et al. 2017; Prăvălie 2016; Yao et al. 2020). These wet‐dry cycles strongly regulate soil microbial activity in drylands, where microbial communities rapidly resuscitate after rainfall pulses (Imminger et al. 2024; Placella et al. 2012). Because microbial metabolism is a major driver of trace gas fluxes from soils (Placella et al. 2012; Schimel et al. 2007), climate‐driven changes in precipitation regimes may therefore alter microbial emissions of reactive gases (Hernandez et al. 2023). Understanding how microbial activation following rainfall events influences atmospheric chemistry is thus critical for predicting biosphere‐atmosphere feedbacks under future climate scenarios.

Biological activity in drylands is tightly coupled with wet‐dry cycles. In the Negev Desert, biocrust‐dominated communities exhibit rapid resuscitation within minutes after rainfall (Imminger et al. 2024), and soil rewetting after drought is a major trigger of microbial metabolism and associated biogeochemical carbon and nitrogen fluxes (Wang et al. 2022). A classic example is the Birch effect, a rapid pulse of CO_2_ released after dry soils are rewetted, which reflects the sudden reactivation of microbial activity and the mobilization of labile substrates that can account for a significant portion of the annual carbon budget (Xu et al. 2004).

Rewetting may also generate significant VOC fluxes. Upon rewetting after prolonged drought, dry soils are known to release a distinctive scent, namely “petrichor” (from Greek *petra*, meaning stone, and *ichor*, the fluid of the gods), a VOC blend typically associated with geosmin and 2‐methylisoborneol (2‐MIB) produced by *Actinomycetota* and filamentous *Cyanobacteriota* (Insam and Seewald 2010). While petrichor is familiar to humans, its detailed chemical composition and the extent to which climate‐ and microbiome‐driven adaptations shape emission rates across drylands remain unknown.

Pulse‐driven dynamics are a defining feature of dryland ecosystems, where episodic rainfall events control microbial activity, carbon turnover, and trace gas fluxes, often accounting for a substantial fraction of annual biogeochemical exchange (Placella et al. 2012). Petrichor can thus be viewed as a biogeochemical pulse: a transient but potentially intense VOC release triggered by soil rewetting, analogous in timing to the CO_2_ Birch effect. Such emissions may reflect both physical desorption or displacement of compounds accumulated during drought and renewed microbial biosynthesis after rehydration. Given the global extent of drylands and the high atmospheric reactivity of many biogenic VOCs (Lelieveld et al. 2008), these short‐lived emissions could transiently influence local OH reactivity, ozone formation, and secondary organic aerosol production. Although rewetting‐induced VOC pulses have been reported from soils and litter (Bourtsoukidis et al. 2018; Crocker et al. 2025; Pugliese et al. 2023; Rossabi et al. 2018), their atmospheric implications remain poorly constrained, particularly for drylands.

Here, we investigated soil VOC emissions and carbon fluxes across a steep aridity gradient in Israel, spanning sub‐humid shrubland outside the Negev Desert and semi‐arid, arid, and hyper‐arid regions within the Negev Desert. These dryland regions differ in climate, soil properties, and microbial community composition (Bay et al. 2021). We simulated two consecutive rainfall events on soils with contrasting drought histories and quantified the chemical composition and temporal dynamics of petrichor alongside soil CO_2_ fluxes under controlled conditions. Upscaling VOC fluxes showed that dryland soils can represent a significant episodic source of reactive VOCs following rewetting. We show that these emissions are shaped by aridity‐dependent microbial community adaptation and may significantly influence regional atmospheric chemistry.

## 2 Results

### 2.1 Soil Bacterial Community Composition Shifts Across the Aridity Gradient

The sub‐humid (SH), semi‐arid (SA), arid (A), and hyper‐arid (HA) regions in Israel differ in climate, soil properties, and microbial community composition (Figure 1, Figures S1 and S2, Data S1, Table S1). Significant changes in bacterial community composition were found in both biocrusts and topsoil across the aridity gradient (Figure 1, Tables S2–S6; *p* = 0.001), with higher within‐region variability in arid zones (PERMDISP, *F* = 11.37, *p* = 0.001). Across both biocrust and topsoil, *Actinomycetota‐*affiliated orders dominated (56%–77%) and increased in relative abundance from sub‐humid toward arid and hyper‐arid regions (Table S3). In biocrusts from semi‐arid (SA) and arid regions, *Cyanobacteriales* peaked at 17%–18%. Additional soil and region‐specific patterns were driven by *Proteobacteria* (14%–22% in biocrust), *Chloroflexota* (9%–14% in topsoil), and *Bacteroidota* and *Verrucomicrobiota* orders as minor contributors. In biocrusts, multiple *Actinomycetota*, *Cyanobacteriales*, and several *Proteobacteria*, *Planctomycetota*, and *Verrucomicrobiota* orders varied significantly among climatic regions (Tables S3–S6; FDR‐corrected *q*‐value < 0.05), whereas topsoil exhibited weaker but overlapping trends. Within each climatic region, many dominant orders differed significantly between biocrust and topsoil, particularly in sub‐humid and arid regions, indicating habitat‐specific selection between surface biocrust and the underlying soil driven by the aridity gradient (Tables S5 and S6).

**FIGURE 1:**
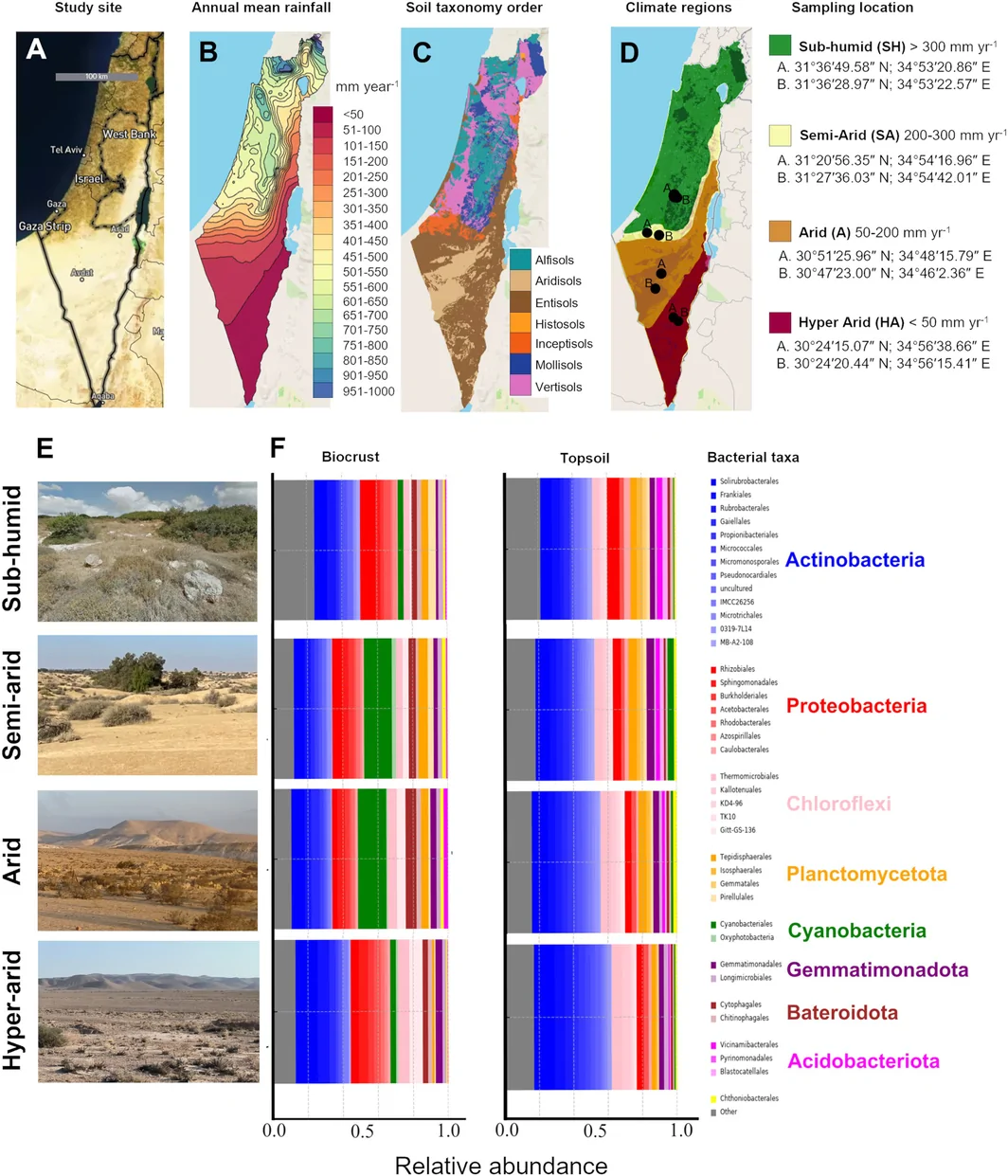
Study sites along the Israel aridity gradient showing climate and soil characteristics, and microbial community composition. (A) Satellite image with country administrative borders (black lines). (B) Annual mean rainfall across the four sampled climatic regions: Subhumid, semi‐arid, arid, and hyper‐arid. (C) Soil taxonomy orders according to USDA classification. (D) Overlay of soil taxonomy orders and mean annual precipitation with the geolocation of sampling sites, showing two spatially separated sites (“A” and “B”) within each climatic region. (E) Photographs of representative collection sites from the sub‐humid, semi‐arid, arid, and hyper‐arid regions. (F) Microbial community composition of biocrust and topsoil samples from each site, based on 16S rRNA gene amplicon sequences. Additional details on precipitation, soil moisture and temperature regimes, and soil taxonomy are provided in Figure S1. Map lines delineate study areas and do not necessarily depict accepted national boundaries.

### 2.2 Petrichor Composition and Emissions Depend on Aridity Gradient and the Succession of Rewetting Events

We quantified the fluxes of VOCs from the soil cores following the two rewetting episodes separated by a month of gradual desiccation, using simulated rewetting experiments and cuvette‐based gas‐exchange measurements (Methods S1–S3, Figures S3–S6, Tables S7–S9). We first used multivariate analyses to identify the main structure of the VOC response across rewetting stages and climatic regions (Figure 2). Rewetting of the soil after 3 months of field drought elicited strong VOC emissions within the first day after the first rewetting, followed by changes in composition and a decrease in emission intensity upon the second rewetting. Petrichor emissions from soils across the climatic regions differed significantly in both intensity and chemical composition during the first and second rewetting episodes, although emissions after the second rewetting were more similar between arid and hyper‐arid regions (Figure 2B,C). No significant differences were detected between different sites within each climatic region (Figure 2A), indicating that emission patterns reflect regional climatic adaptation rather than local variability. Across all soils, we detected significant emissions of 58 VOCs, regardless of soil origin. Several benzenoid compounds were strongly associated with immediate emissions and CO_2_ flux after the first rewetting, whereas several terpenoids were characteristic of the second rewetting event (Figure 2D–F, Figure S7, Table S8). Geosmin, a characteristic volatile often identified with petrichor, was also detected in the blend of volatiles after rewetting (Figure S8). However, its emission rate was low (< 1 pmol m^−2^ s^−1^) compared to total terpenes (210–640 pmol m^−2^ s^−1^), benzenoids (187–634 pmol m^−2^ s^−1^), and other VOCs (1050–3890 pmol m^−2^ s^−1^), which were mainly composed of the carbonyl compounds acetaldehyde, acetone, and acetic acid, emitted by all soil samples (Figure 2G–I). No significant emissions of 2‐MIB were detected.

**FIGURE 2:**
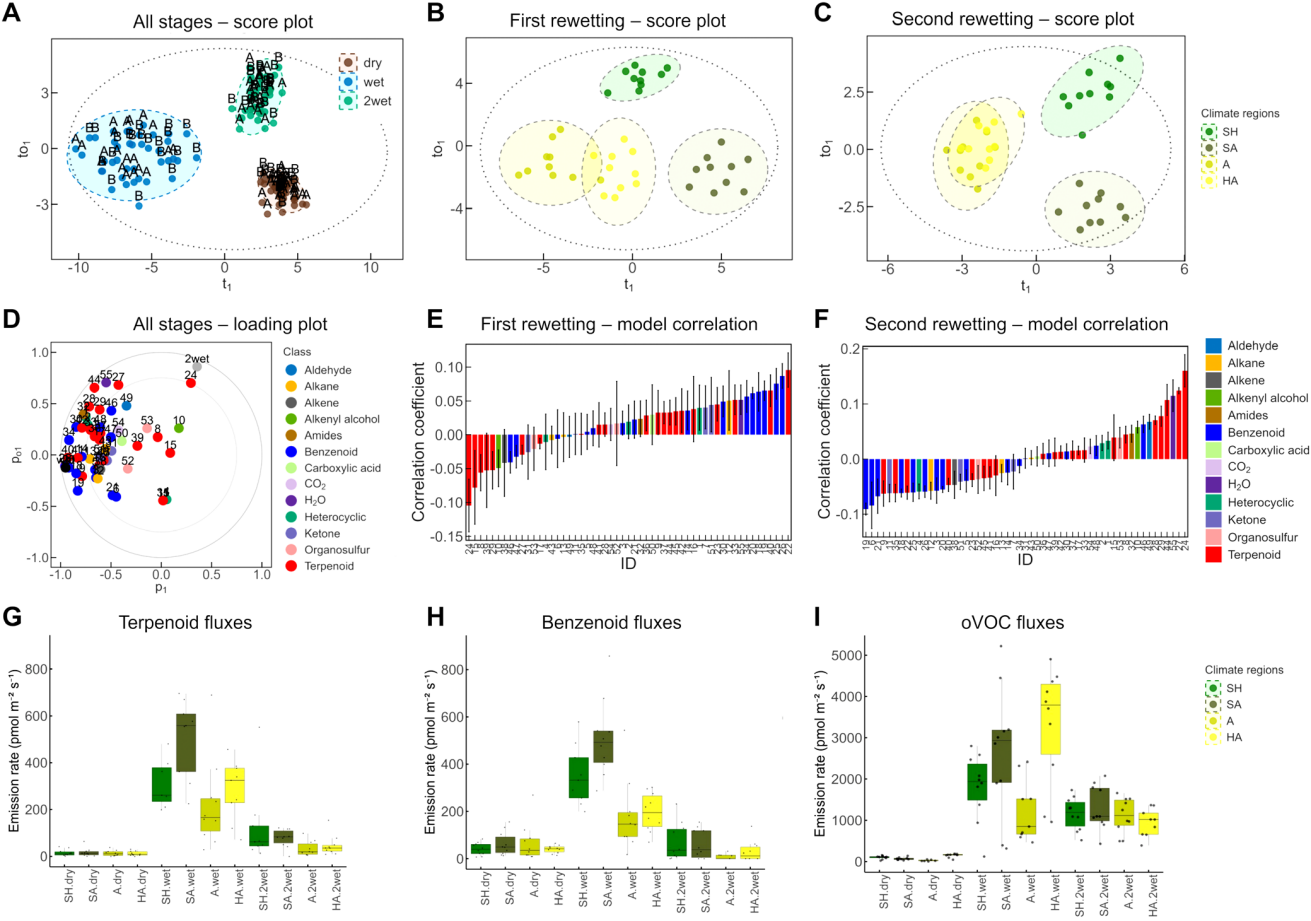
Changes in VOC emissions after two consecutive soil rewetting events. Orthogonal partial least squares (OPLS) analysis of VOC emission rates from soils collected from four climatic regions: Sub‐humid (SH), semi‐arid (SA), arid (A), and hyper‐arid (HA). VOC fluxes were measured under dry conditions and following two consecutive rewetting events separated by a 31‐day desiccation period. (A) OPLS score plot including all samples and sampling stages; score plots for VOC fluxes after (B) the first rewetting and (C) the second rewetting (OPLS model correlation plots are shown in Figure S9). (D) Normalized and correlation‐scaled loading plot corresponding to the score plot of the complete dataset. Each numbered point represents an individual VOC, listed in Table S7. The horizontal (*p1*) and vertical (*po1*) positions indicate the association of each VOC with the predictive and orthogonal variation, respectively, underlying the sample separation shown in the score plot. VOCs farther from the origin have stronger associations with the corresponding model components, whereas VOCs close to the origin show weaker associations. VOCs located in similar directions show similar patterns across samples, whereas those positioned in opposite directions show contrasting patterns. (E, F) OPLS model correlation coefficients identifying VOCs associated with the first rewetting relative to the dry and second‐rewetting stages (E), and with the second rewetting relative to the dry and first‐rewetting stages (F). Positive coefficients indicate VOCs enriched during the respective rewetting event, whereas negative coefficients indicate lower fluxes relative to the remaining stages. VOC IDs correspond to compound names listed in Table S7. Box plots show emission rates for (G) total terpenoids, (H) benzenoids, and (I) oxygenated small VOCs (oVOCs; acetaldehyde, acetone, and acetic acid). Each point represents the 17‐h integrated flux of one soil‐core replicate. For each climatic region, *n* = 10 soil cores were collected from two distal sites, 2–10 km apart (“A” and “B” in panel A). In the score plots (A–C), colored ellipses indicate 95% confidence intervals around group centroids, and the dotted outer ellipse represents the Hotelling's *T* ^*2*^ 95% confidence region. All OPLS models were statistically significant (CV‐ANOVA: A, *p* < 1 × 10^−10^; B, *p* = 1 × 10^−8^; C, *p* = 1 × 10^−5^). Model fitness: A, R^2^X(cum) = 0.59, R^2^Y(cum) = 1, Q^2^Y(cum) = 0.87; B, R^2^X(cum) = 0.63, R^2^Y(cum) = 1, Q^2^Y(cum) = 0.76; C, R^2^X(cum) = 0.68, R^2^Y(cum) = 1, Q^2^Y(cum) = 0.53.

We next tested whether rewetting‐induced VOC emissions varied systematically along the aridity gradient. OPLS models constructed separately for the first and second rewetting events showed that aridity significantly shaped petrichor emissions in both cases (*p* < 0.001, CV‐ANOVA; Figure 3), affecting both VOC composition and emission fluxes. Lower prediction errors after the first rewetting indicate that the initial VOC pulse carried the strongest aridity‐related signature. Terpenoid‐rich emissions were characteristic of the wetter sub‐humid region, particularly monoterpenoids such as eucalyptol and camphor (Figures 3, S9, S10, Table S8). In contrast, benzenoids such as xylenes and indane, together with the sesquiterpene longicyclene were associated with the semi‐arid region, whereas benzenoids and the monoterpenoids 2‐methyl‐2‐bornene and (*E*)‐p‐menthan‐1‐ol were linked to the arid region. Emissions of isolongifolene, acetaldehyde, and alkanes (e.g., decane) were positively correlated with the hyper‐arid region. After the second rewetting, the aridity effect persisted but shifted mainly toward differences in mono‐ and sesquiterpenoid emission profiles (Figure 3F,H). Overall, the availability of C, N, P, Fe, and Mn was higher in the wetter sub‐humid and semi‐arid regions, whereas soluble ions (e.g., NO_3_^−^, Cl^−^, Na^+^) were significantly higher in the drier arid and hyper‐arid regions (Figure S2).

**FIGURE 3:**
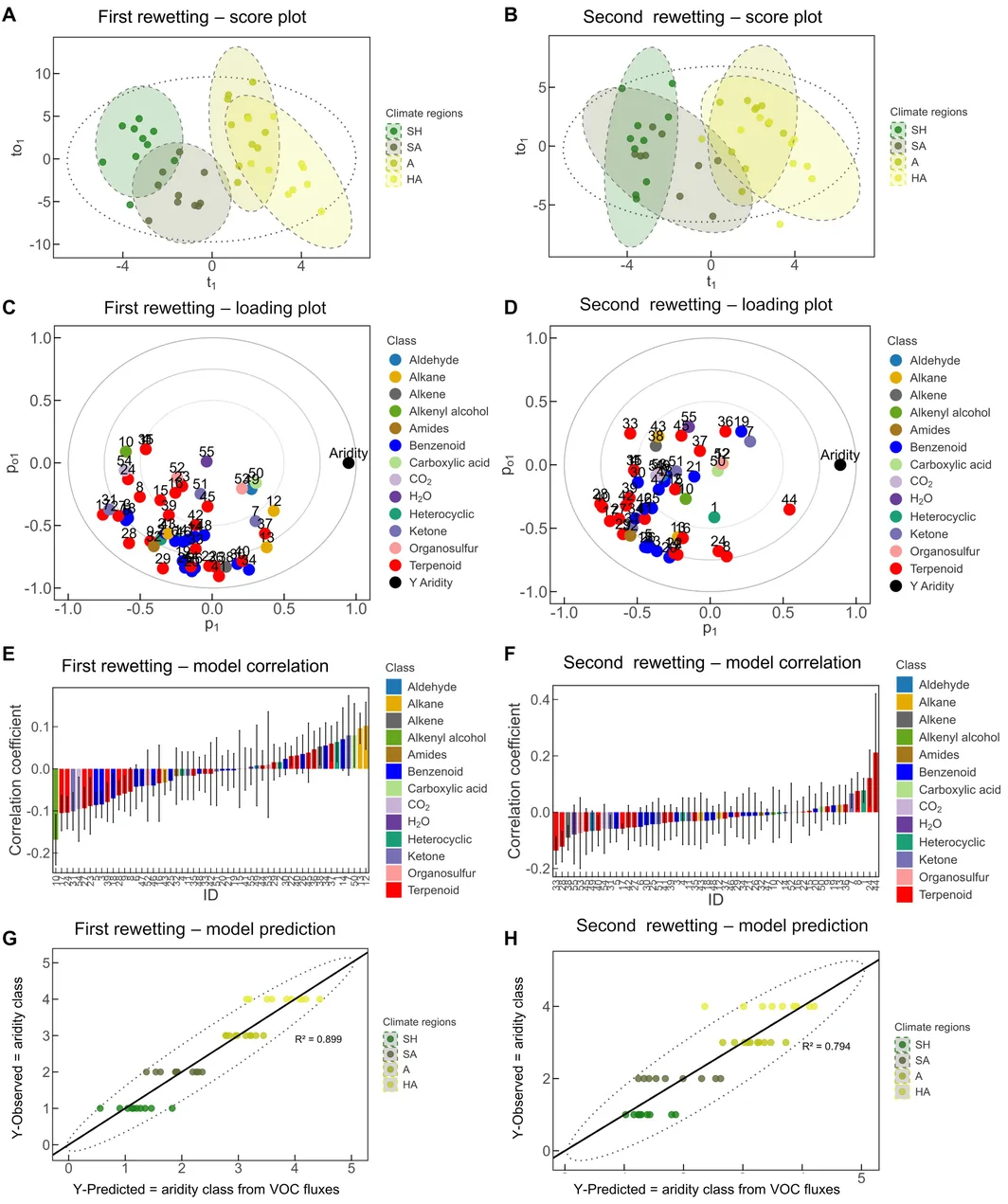
Aridity‐dependent differences in VOC emissions after soil rewetting. OPLS analysis of VOC fluxes showing the relationship between petrichor emissions and aridity after the first rewetting event (left panels) and second rewetting event (right panels). Climatic regions are sub‐humid (SH), semi‐arid (SA), arid (A), and hyper‐arid (HA). (A, B) OPLS score plots; (C, D) Normalized and correlation‐scaled loading plots corresponding to the OPLS models shown in A and B. Each numbered point represents an individual VOC, listed in Table S7. The horizontal predictive loading (*p*1) describes the variation in VOC emissions associated with the aridity gradient, whereas the vertical orthogonal loading (*p*o1) represents systematic VOC variation unrelated to aridity. The black point represents the response Y‐variable (Aridity). VOCs positioned in the same direction along *p*1 as the Aridity variable are positively associated with increasing aridity, whereas VOCs positioned in the opposite direction are negatively associated. Larger absolute loadings indicate stronger associations with the respective model components. (E, F) OPLS model correlation coefficients identifying VOCs associated with the aridity gradient after the first rewetting (E) and second rewetting (F). Positive coefficients indicate VOCs enriched with increasing aridity, whereas negative coefficients indicate VOCs enriched toward less arid climatic regions. (G, H) Predicted vs observed aridity class for the first rewetting model (G; *p* < 1 × 10^−9^) and second rewetting model (H; *p* = 1.13 × 10^−4^, CV‐ANOVA); climatic regions were encoded as an ordered aridity Y‐variable from low to high aridity: Sub‐humid (Y = 1), semi‐arid (Y = 2), arid (Y = 3), and hyper‐arid (Y = 4). The y‐axis shows the observed aridity class of each sample, whereas the x‐axis shows the aridity class predicted from the VOC emission profile by the OPLS model. Root mean square errors of estimation and cross‐validation were RMSEE = 0.375 and RMSEcv = 0.537 for the first rewetting model, and RMSEE = 0.533 and RMSEcv = 0.809 for the second rewetting model. In the score plots (A, B), the dotted outer ellipse represents the Hotelling's *T* ^*2*^ 95% confidence region, while colored ellipses indicate 95% confidence intervals around climatic‐region centroids. Model fitness: A, R^2^X(cum) = 0.53, R^2^Y(cum) = 1, Q^2^Y(cum) = 0.90; B, R^2^X(cum) = 0.32, R^2^Y(cum) = 1, Q^2^Y(cum) = 0.79. Numbers in C‐F indicate the VOC IDs listed in Table S7.

Together, these results show that petrichor is not a uniform rewetting response across drylands, but an aridity‐dependent VOC pulse whose intensity and chemical composition vary systematically along the climatic gradient.

### 2.3 Emission Dynamics and Correlations of VOCs, H_2_O, and CO_2_ Fluxes

Rewetting of arid soils is known to induce sharp CO_2_ pulses, yet the temporal dynamics of VOC emissions during these events remain unknown. To capture these rapid metabolic and physicochemical shifts, we simultaneously measured real‐time fluxes of VOC, H_2_O, and CO_2_ from the soil cores over four consecutive days after each rewetting (Figure 4). As expected, rewetting caused a rapid and pronounced pulse of CO_2_ (the Birch effect). Notably, VOC emissions showed temporal dynamics similar to CO_2_, with acetaldehyde showing the strongest similarity (Figure 5).

**FIGURE 4:**
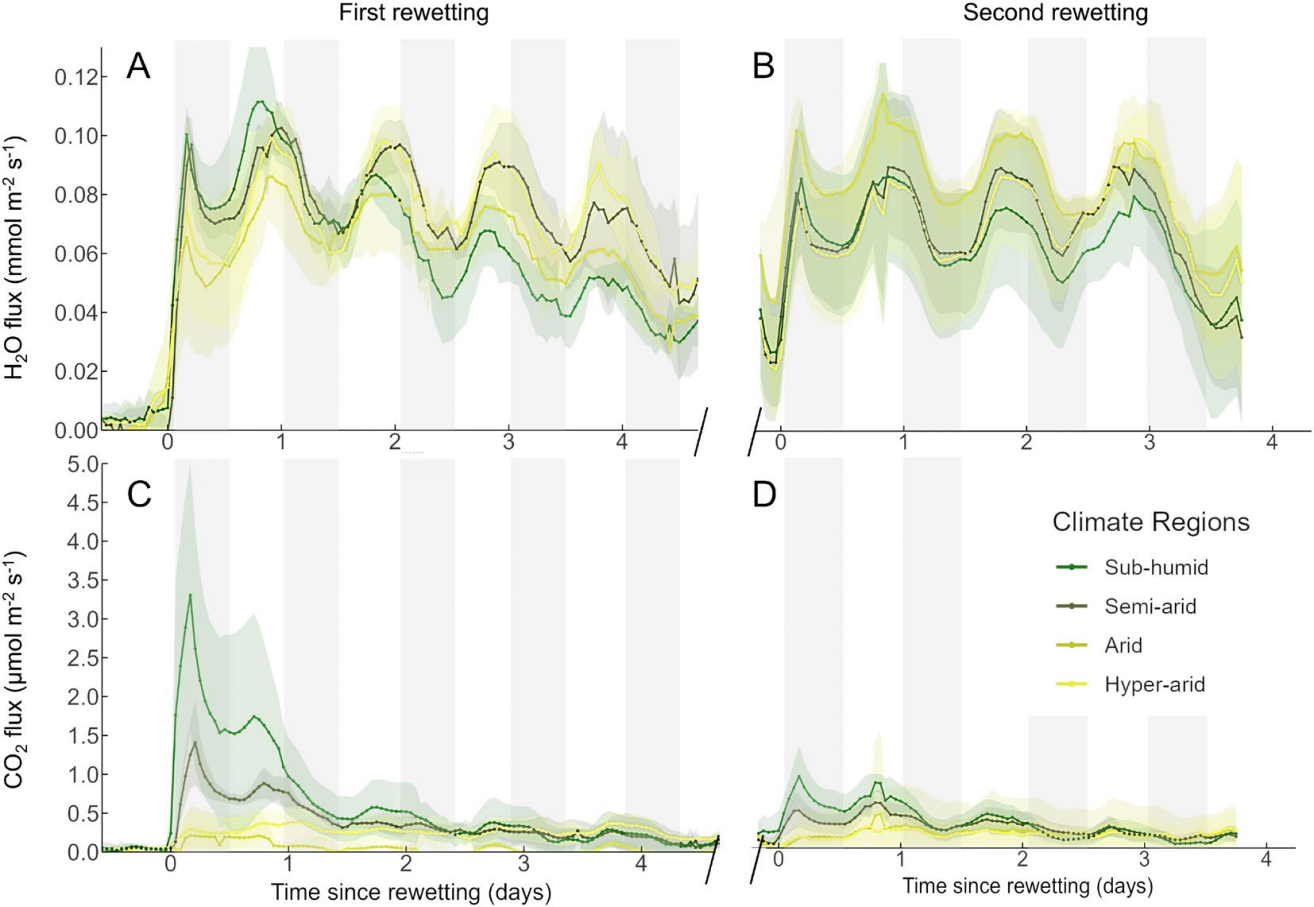
Soil H_2_O and CO_2_ flux responses to two rewetting events across an aridity gradient under controlled environmental conditions. Mean fluxes of H_2_O (A, B) and CO_2_ (C, D) measured in soils from the four climatic regions. Left and right panels refer to the first and second rewetting events, respectively. After the first rewetting, the soils were allowed to dry for 31 days under standard conditions (T = 23°C ± 0.4°C; RH 53% ± 5%). Time is expressed relative to the onset of rewetting (Day 0), and the day/night cycle is indicated with white/grey bars. Lines represent mean fluxes and shaded areas represent the 95% confidence intervals (*n* = 10).

**FIGURE 5:**
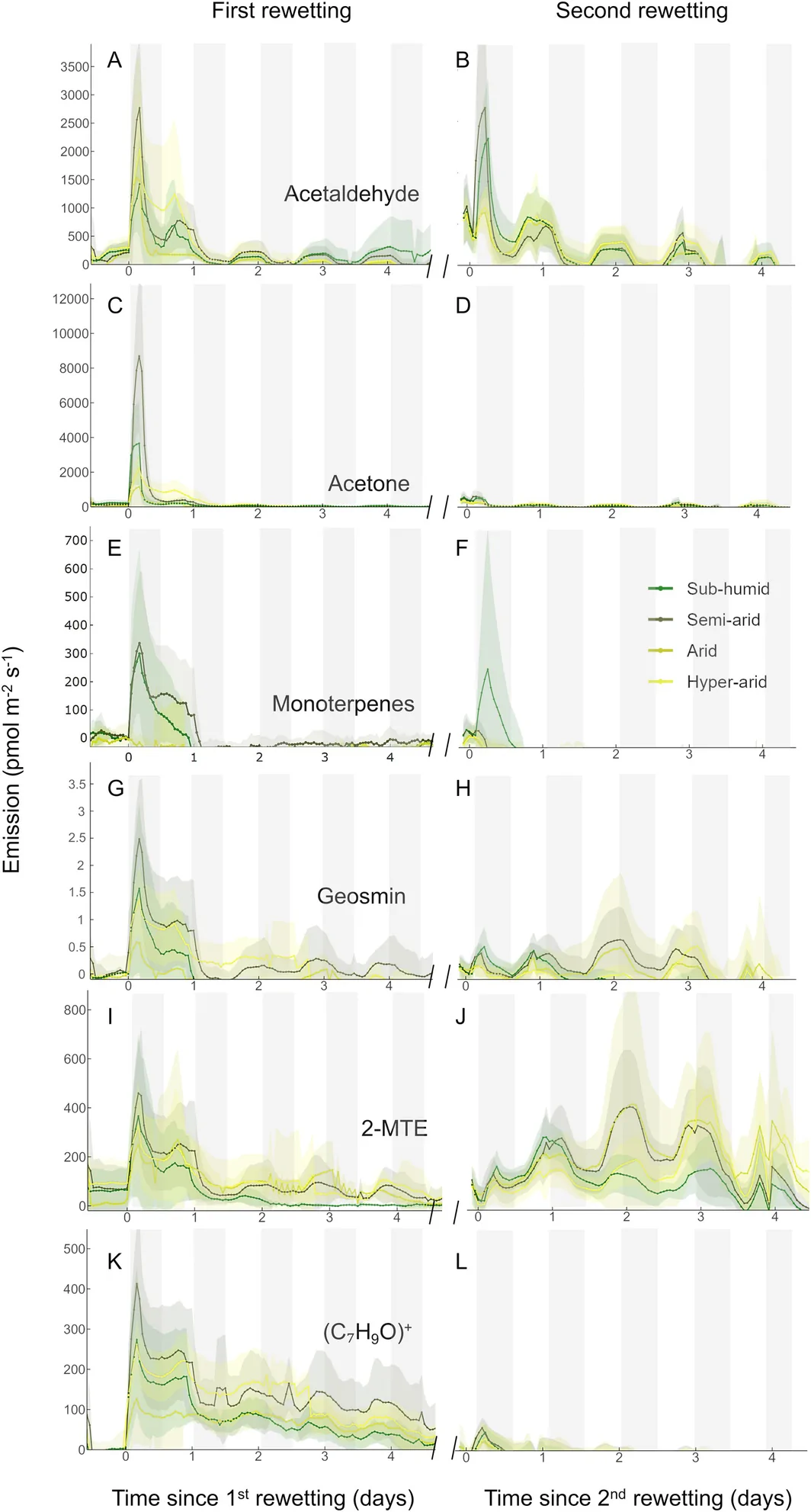
Emission dynamics of VOCs in the petrichor in response to two rewetting events. Time series of VOC emission rates (normalized to ground area) measured by PTR‐MS for acetaldehyde (A, B), acetone (C, D), monoterpenes (E, F), geosmin (G, H), 2‐methylthioethanol (2‐MTE) (I, J), benzenoid and aromatic alcohols at C_7_H_9_O+ (K, L). Left and right panels refer to emissions following the first and second rewetting events, respectively, separated by 31 days of desiccation. Soil cores originated from the four climatic regions: Sub‐humid, semi‐arid, arid, and hyper‐arid. Time is expressed relative to the onset of rewetting (Day 0), and the day/night cycle is indicated with white/grey bars. Lines represent mean fluxes and shaded areas represent the 95% confidence intervals (*n* = 10).

Water vapor fluxes across the aridity gradient were essentially similar after the first and second rewetting events (Figure 4A,B), reflecting comparable evaporation under controlled conditions despite differences in soil texture (Figure S2; Table S2). Conversely, fluxes of CO_2_ were much higher after the first rewetting event following drought and then declined with the second rewetting, suggesting that some labile carbon pools were depleted after a short desiccation‐hydration cycle. When comparing the soils, CO_2_ fluxes were significantly higher from soils collected in regions with higher precipitation (the sub‐humid and semi‐arid regions) compared to the drier arid and hyper‐arid regions (Figure 4). The day/night cycles affected both the H_2_O and CO_2_ fluxes through changes in temperature and relative humidity inside the cuvettes [28.4/22.8°C, 11/65% RH (light/dark), Figure S4]. Importantly, CO_2_ fluxes were nearly zero before both rewetting episodes (dry conditions), indicating that the microbial metabolic activity was strongly suppressed by desiccation (Figure 4C,D).

VOC emissions generally peaked within hours of rehydration, exhibiting distinct temporal profiles and intensities across compound classes and between wetting events (Figure 5). Acetaldehyde emissions were rapidly and strongly induced after the first rewetting (1000–3000 pmol m^−2^ s^−1^, peaking at 3–4 h after rewetting), with comparable peak magnitudes following the second rewetting (Figure 5A,B). In contrast, acetone emissions were markedly higher during the first rewetting (up to 8000 pmol m^−2^ s^−1^, peaking at 3–4 h after rewetting) but declined significantly during the second event (220–580 pmol m^−2^ s^−1^) (Figure 5C,D). Larger ketones (C_4_–C_10_) followed a similar trend (Figure S11). Acetic acid mirrored acetaldehyde, decreasing from the first to the second rewetting events, but the reduction was smaller than that of ketones. Terpene emissions were strongly elicited upon rewetting (Figure S12). Monoterpene emissions were particularly associated with soils from the sub‐humid regions (Figure 5E,F), which contained small particles of plant material (Figure S6). Geosmin emissions from the semi‐arid region increased gradually over time following an initial transient pulse and remained consistently higher than those from other soil types throughout the measurement period (Figure 5G,H). In contrast, monoterpenoid emissions were low during the second rewetting event and were primarily associated with sub‐humid and semi‐arid soils.

The sulfurous volatile compounds dimethyl sulfide (DMS) and 2‐methylthioethanol (2‐MTE) exhibited contrasting emission patterns across wetting events. A pronounced DMS burst was observed following the first rewetting, particularly in soils from the sub‐humid region (Figure S13). In contrast, 2‐MTE emissions increased notably after the second rewetting (Figure 5I,J), with the highest fluxes observed in soils from the semi‐arid and arid climatic regions. Benzenoid and aromatic alcohol compounds, detected at m/z 109 [(C_7_H_9_O)^+^], and xylenes (m/z 107), showed a rapid decline over time (Figure 5K,L; Figure S13). Although the second rewetting induced a temporary emission, levels dropped quickly and became undetectable within 2 days.

Diurnal cycles in VOC fluxes were evident for several other compounds (e.g., MT, geosmin, and aldehydes), suggesting a strong influence of temperature and light on emission dynamics (Figures 5, S4, S11–13). The emission dynamics of acetaldehyde, acetone, acetic acid, and 2‐MTE were strongly interrelated (clustered heatmap, Figure S14). In particular, acetaldehyde and acetic acid showed strong correlations (Spearman's correlation coefficients > 0.91). When comparing individual volatiles measured by GC–MS and PTR‐MS, emissions of several terpenoids and benzenoids were also found to correlate (Figure S15), suggesting a common microbial source or shared biochemical origins.

### 2.4 Petrichor Emissions Impact OFP and SOAP

To assess the potential impact of the strong petrichor VOC emissions on atmospheric chemistry, we calculated the ozone‐forming potential (OFP) and secondary organic aerosol potential (SOAP) expected after the first rewetting event (Figure 6). While OFP and SOAP provide more meaningful metrics than mass emissions by accounting for VOC reactivity and SOA yields, respectively, actual production rates depend on nonlinear chemistry involving NOx, sunlight, and environmental conditions that vary with location and time (Wang et al. 2019). Mass emissions, OFP, and SOAP (g m^−2^ day^−1^) were calculated for each compound, summed by chemical category (benzenoids, monoterpenoids, sesquiterpenoids, other), and determined separately for each soil and climatic region. The area‐specific emission rates across the aridity gradient were then upscaled to land areas to estimate total petrichor VOC emissions for Israel, yielding an estimate of 400 metric tons (MT) per day. The petrichor OFP and SOAP estimates were 1120 MT day^−1^ (55 kg km^−2^ day^−1^) for OFP and 27.6 MT day^−1^ (1.36 kg km^−2^ day^−1^) for SOAP.

**FIGURE 6:**
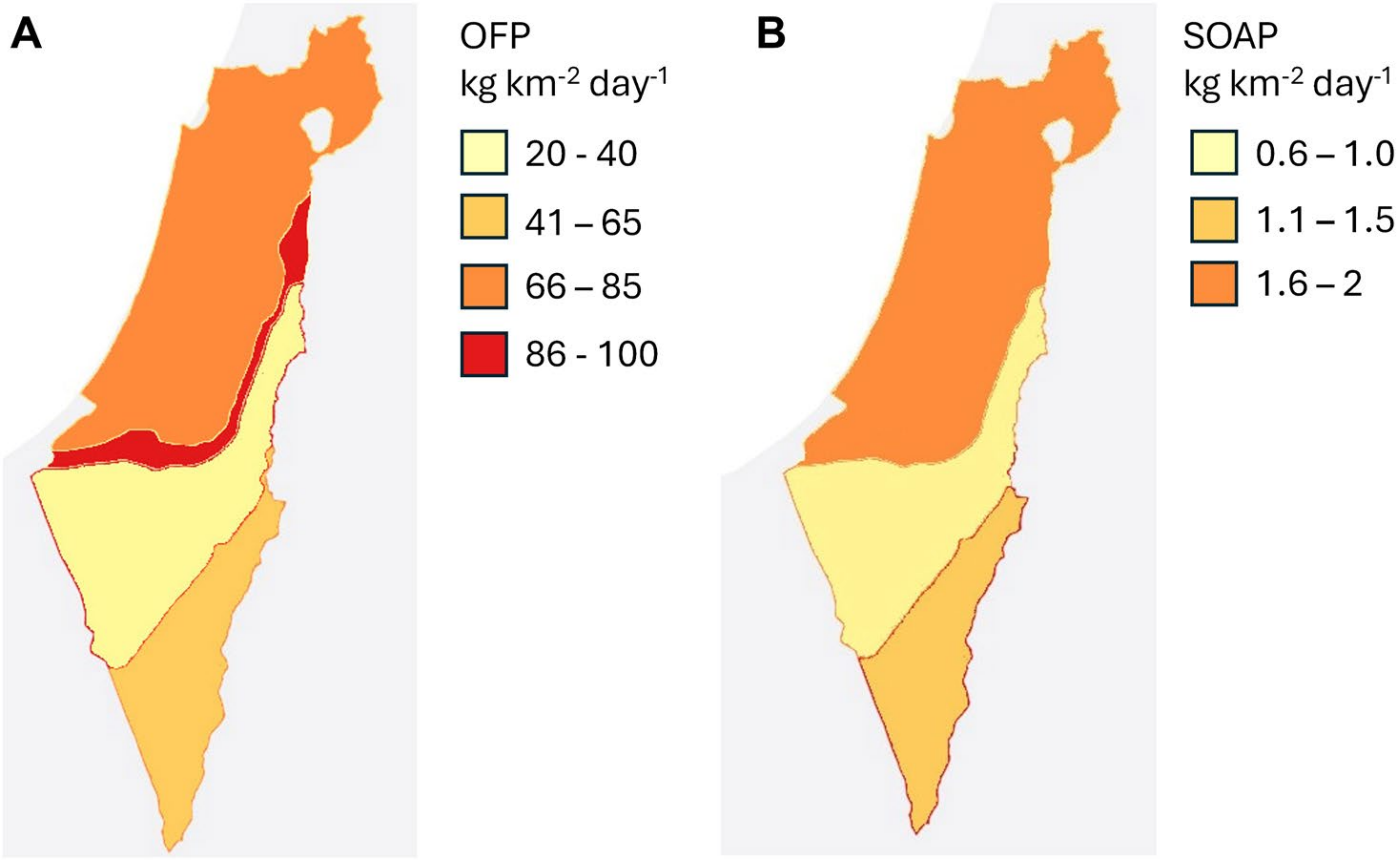
Petrichor‐driven O_3_ and SOA formation potential in Israel. (A) OFP and (B) SOAP were calculated from climate‐region‐specific petrichor VOC fluxes from soils measured the day after rewetting, using maximum incremental reactivity (MIR) and SOAP weighting factors for each compound. The figures show OFP and SOAP formation associated with post‐rewetting petrichor emissions across regions. Map lines delineate study areas and do not necessarily depict accepted national boundaries.

Although the semi‐arid region had > 40% higher mass emissions than the sub‐humid region, its OFP was only 14% higher and its SOAP was similar (−1%) (Figure S16). The hyper‐arid region mass flux was only 11% lower than in the sub‐humid region, while the OFP and SOAP were about 30% lower. In both cases, the differences between the mass flux and the OFP/SOAP results were due to the relatively high contribution of monoterpenes to the sub‐humid flux. In contrast, the arid region fluxes were consistently ~58% lower than in the sub‐humid region for mass, OFP, and SOAP due to their similar composition.

## 3 Discussion

### 3.1 Strong VOC Fluxes Upon Rewetting After Drought

Rewetting dry soils after prolonged drought across a steep aridity gradient triggered strong pulses of VOC emissions, analogous in timing to the CO_2_ Birch effect observed for soil respiration after rewetting (Birch 1958; Xu et al. 2004). Cross‐study comparisons are limited by differences in measurement systems, inlet‐air composition, soil moisture history, and the limited overlap in reported VOCs. Nevertheless, the magnitude of the dryland pulses observed here was comparable to, or greater than, wetting‐ or rainfall‐induced VOC emissions reported from wetter ecosystems. For example, our dryland soils emitted acetaldehyde and DMS in the range of 480–1813 and 21–380 pmol m^−2^ s^−1^, respectively, overlapping with or exceeding rewetting‐induced pulses reported from experimental rainforest soils (Pugliese et al. 2023). In Amazonian soils, Bourtsoukidis et al. (2018) reported higher sesquiterpene and acetone fluxes after rainfall, whereas monoterpene fluxes were near zero in that system (Bourtsoukidis et al. 2018); in contrast, our dryland soils showed comparatively strong monoterpene and benzenoid emissions. Rossabi et al. (2018) further showed that wetting can induce chemically diverse VOC pulses across agricultural, grassland, and forest soils, supporting the idea that rewetting‐induced VOC release is a recurrent response across ecosystems.

These rewetting‐induced emissions contrasted strongly with dry pre‐rewetting conditions, where terpenoid fluxes were negligible, DMS was not detected, and acetaldehyde and acetone remained low. Thus, dry soils generally showed weak VOC emissions before rewetting, whereas rewetting after prolonged drought triggered large, compound‐specific VOC pulses. Our measurements represent rewetting‐induced VOC emission potentials under nearly VOC‐free inlet air, rather than in situ net soil‐atmosphere exchange. This approach minimized background interference but does not capture potential VOC uptake under ambient conditions when concentrations exceed compound‐specific compensation points (Jiao et al. 2023; Rinnan and Albers 2020). However, background biogenic VOC concentrations, and therefore potential uptake, in sparsely vegetated drylands are likely low before rain events, whereas VOC accumulation in the cuvette headspace after soil rewetting may reduce the soil‐air concentration gradient and dampen measured emissions of some compounds.

Nevertheless, the VOC bursts observed after rewetting likely reflect both abiotic and biotic processes, including desorption or displacement of compounds accumulated during drought, osmotic‐shock‐induced cell damage, rapid mineralization of labile organic matter, and microbial metabolic activation (Bear and Thomas 1966; Insam and Seewald 2010; Schimel et al. 2007). The relative contribution of passive release and renewed microbial activity is discussed in more detail below. Overall, rewetting‐induced VOC pulses may be a recurring feature of soil biogeochemical responses across biomes, but their magnitude, chemical composition, and atmospheric relevance remain particularly underexplored in drylands.

### 3.2 Microbial and Physicochemical Controls on Petrichor Emissions

Petrichor composition differed across the aridity gradient, reflecting differences in climatic history, water availability, soil organic matter, and microbial communities (Figure 1). Yet, the same core of VOCs was detected across climatic regions, albeit in different proportions. Aldehydes, ketones, benzenoids, and terpenoids dominated the first rewetting pulse (Figures 2, S11), consistent with microbial stress responses, rapid carbon processing, and byproduct release under low‐oxygen conditions in rewetted arid topsoil (Insam and Seewald 2010; Wheatley 2002). The emission dynamics likely reflect coupled biochemical and physicochemical processes involving metabolites that accumulate during drought and are subsequently released upon soil rewetting, either from damaged microbial cells or from soil pores (Hernandez et al. 2023; Pugliese et al. 2023; Schimel et al. 2007). During desiccation, microbes accumulate osmolytes to maintain cellular integrity, and upon rewetting, the abrupt drop in osmotic pressure promotes cell lysis, osmolyte release, and rapid microbial resuscitation (Imminger et al. 2024; Schimel et al. 2007). Released osmolytes and labile organic substrates, including sugars, amino acids, and other compounds, can be rapidly catabolized, producing CO_2_ and oxygenated VOCs such as acetate, acetone, aldehydes, alcohols, and small organic acids (Fall 2003; Hernandez et al. 2023). In parallel, physicochemical processes also cause VOC emissions (Insam and Seewald 2010). During drought, organics are adsorbed in soil micropores, and when rewetted, changes in gas diffusion, sorption equilibria, and soil polarity promote desorption and volatilization, leading to a rapid VOC pulse (Schimel et al. 2007). Recent VOC time‐series studies suggest that rapid wetting‐induced peaks often reflect physical release or desorption, whereas delayed or sustained emissions indicate renewed microbial activity (Crocker et al. 2025; Kim et al. 2021). Accordingly, the rapid increase in oxygenated VOCs may partly reflect passive release after rewetting, while sustained or secondary increases in terpenoids and sulfur‐containing VOCs suggest active microbial contributions, consistent with microbial secondary metabolism, isoprenoid biosynthesis, and sulfur metabolism (Insam and Seewald 2010; Juttner and Watson 2007; Schulz and Dickschat 2007).

Together, moisture‐driven physicochemical release, microbial lysis, and rapid microbial resuscitation likely explain the strong petrichor pulse observed after rewetting across the aridity gradient. However, temporal emissions alone cannot conclusively distinguish *de novo* microbial production from physicochemical release, which would require stable isotope labeling studies or pathway‐specific molecular evidence. Nevertheless, compound‐specific dynamics, their coupling to CO_2_ fluxes, and their systematic variation across the aridity gradient support an important microbial contribution to sustained petrichor emissions.

The dynamics of benzenoid emissions likely reflect a combination of rapid desorption from soil surfaces and microbial production linked to the shikimate pathway. Some benzenoids (e.g., ethenylbenzene) are known to originate from microbial phenylpropanoid metabolism, especially in Proteobacteria and fungi (Choudoir et al. 2019; Garcia‐Franco et al. 2024). However, hydrophobic aromatics such as xylenes, ethyl‐ and polymethyl‐benzenes, and indanes may partly derive from anthropogenic combustion residues that are transported over long distances on black‐carbon particles, which are abundant in dryland soils and exhibit a strong affinity for aromatic hydrocarbons (Glaser et al. 1998; Kwon and Pignatello 2005). Rewetting alters sorption equilibria and pore accessibility, enabling desorption, as discussed above. This likely explains the strong attenuation of these emissions upon a second rewetting under standardized conditions.

Terpenoids also peaked immediately after rewetting from soils of the sub‐humid regions, but they also increased over time. Terpenoid biosynthetic genes are widespread in soil bacteria and fungi, acting as cell membrane stabilizers, oxidative protectants, and infochemicals mediating microbial communication and competition (Crits‐Christoph et al. 2018; Tyc et al. 2017). Their delayed appearance suggests linkage to microbial growth phases and de novo processes. The initial emission pulse likely reflects the physical release of preexisting terpenoids associated with the producer cell membranes and/or stored pools that accumulated during desiccation, while the subsequent gradual increase suggests de novo biosynthesis during the microbial regrowth phase. Rewetting stimulates microbial resuscitation (Angel and Conrad 2013), which can lead to renewed metabolic activity in terpene‐producing genera such as *Streptomyces* (*Actinomycetota*), *Collimonas* (Proteobacteria), and filamentous fungi (Tyc et al. 2017). As these organisms shift from maintenance to growth, carbon flow through terpene pathways may increase. The delayed increase in terpenoid emissions may therefore reflect microbial succession and metabolic recovery rather than purely abiotic desorption. This interpretation is consistent with the time scales of rapid resuscitation and secondary metabolism following rewetting (Bay et al. 2021; Imminger et al. 2024), and could be tested using stable isotope approaches. While we cannot fully disentangle microbial biosynthesis from physicochemical release, the compound specificity, temporal evolution, and coupling to CO_2_ fluxes and microbial community structure indicate a dominant microbial control on sustained emissions.

Microbial sulfur volatiles such as DMS and 2‐MTE are important mediators of plant‐microbe and microbe‐microbe interactions (Tyc et al. 2017). Interestingly, we observed contrasting dynamics of sulfur VOCs: DMS peaked immediately after the first rewetting, possibly reflecting stress‐related release, while 2‐MTE dominated after the second wetting (Figures 5 and S13), suggesting slower microbial sulfur metabolism. Similar sulfur pulses have been observed in rainforest rewetting experiments (Pugliese et al. 2023). Metabolic production of 2‐MTE is intriguing, as it is a product of sulfur recycling by soil bacteria that possess a methylthio‐alkane reductase (North et al. 2020). Some bacteria can use 2‐MTE to produce methionine and S‐adenosylmethionine under limited sulfur conditions and in anoxic environments (North et al. 2020), a strategy to scavenge sulfur in anaerobic soil niches. Such anoxic microsites have been documented in rewetted biocrusts and desert soils (Maier et al. 2021). Yet, linking these dynamic sulfur signals to functional expression will require targeted omics and mechanistic studies.

### 3.3 Microbial Community and Aridity Gradient Effects

Soil microbial community composition shifted strongly across the aridity gradient (Bay et al. 2021) (Figure 1). The bacterial community profiles were obtained from a previous dry‐season sampling effort at the same sites (Bay et al. 2021); therefore, we cannot exclude the possibility that fine‐scale soil heterogeneity contributed to differences in community composition and measured emission potentials. Nevertheless, these profiles provide a representative dry‐season, site‐level microbial context, supported by previous long‐term observations showing stable microbial community patterns across this aridity gradient during drought conditions (Angel et al. 2010; Bachar et al. 2010; Bay et al. 2020, 2021).

Sub‐humid and semi‐arid soils showed higher CO_2_ fluxes, reflecting greater total organic‐carbon content, whereas arid and hyper‐arid soils emitted proportionally more geosmin and sulfur volatiles, consistent with the dominance of stress‐tolerant, trace gas‐oxidizing *Actinomycetota* taxa (Bay et al. 2021). These trends align with metagenomic evidence that desert communities rely increasingly on chemosynthetic and hydrogenotrophic pathways under extreme aridity (Maier et al. 2021).

As the aridity gradient also implies shifts in nutrient availability (C, N, and P) and salinity (Figure S2), these chemical differences likely further shaped the volatilome. Nutrient‐richer sub‐humid soils appear to promote *de novo* microbial VOC biosynthesis during post‐wetting regrowth, whereas nutrient‐poor, saline arid and hyper‐arid soils showed VOC signatures consistent with osmotic‐shock responses, characterized by early emission pulses dominated by low‐molecular‐weight carbonyls, alkanes, and benzenoid compounds (Insam and Seewald 2010; Schimel et al. 2007).

We hypothesize that the VOC emissions help shape the microbial community structure, as many microbial volatiles function as allelochemicals that inhibit competitors or modulate growth and survival (Schmidt et al. 2015). Microbial volatiles can alter gene expression and modulate physiology and biofilm formation in VOC‐exposed bacteria (K. S. Kim et al. 2013). The microbial communities resuscitate within minutes of hydration in these drylands, allocating much of their early activity to cellular repair, energy generation, and competitive interactions (Imminger et al. 2024; Schmidt et al. 2015). The climate‐specific and temporal shifts in VOC composition observed here may serve as fingerprints of microbial community structure, physiological activity, stress responses, and interspecific communication, yet their ecological roles in desert soils remain to be discovered. These volatile signals likely act at short and large spatial scales, with unclear broader ecological consequences (Melo et al. 2020; Wood et al. 2022; Zaroubi et al. 2022).

### 3.4 Atmospheric Impact of Soil VOC Emissions From Drylands

Petrichor VOCs include highly reactive compounds with potential atmospheric relevance. Benzenoids and alcohols may act as sinks for hydroxyl radicals, while emissions of terpenoids serve as precursors for SOA and O_3_ formation and contribute to OH recycling (Lelieveld et al. 2008; Machado et al. 2024). DMS is a key biogenic sulfur gas that affects aerosol and cloud formation (Charlson et al. 1987). Thus, both the magnitude and the chemical composition of desert soil VOC fluxes are important for evaluating their atmospheric impact.

Quantitatively, using the daily mean emission rates, we estimated a significant total petrichor VOC emission of ~20 kg km^−2^ day^−1^, of the same order of magnitude as Israel's reported anthropogenic VOC emissions (~14.4 kg km^−2^ day^−1^) (Muntean et al. 2018). The ozone formation potential (55 kg km^−2^ day^−1^) and SOA formation potential (1.36 kg km^−2^ day^−1^) associated with petrichor emissions are of a similar magnitude to, although approximately 25% lower than anthropogenic VOC emissions in a highly urbanized area such as Los Angeles (Gu et al. 2021). Compared to Los Angeles's urban forest biogenic VOCs, petrichor VOCs show lower ozone formation but higher SOA formation, due to the dominance of monoterpenoids rather than isoprene. Even without accounting for ~6% built‐up land cover (~8%–9% including inland water bodies) in Israel, these comparison values indicate that petrichor emissions from Israel may contribute to local atmospheric chemistry. The ultimate impact on oxidants and aerosols will depend on ambient NO_x_ levels. In Israel, surface and satellite observations show pronounced temporal variability in NOx, including seasonal, weekly, and diurnal cycles linked to emissions and photochemical loss, with mean annual values ranging from ~10 to > 35 ppb in rural and suburban areas (Boersma et al. 2009). Local combustion sources can also strongly enhance NO and NOx, as shown by monitoring studies near transport corridors in central Israel, with concentrations ranging from 25 to > 300 ppb (Tartakovsky et al. 2025). The approach used here to calculate OFP values is based on scenarios optimized for high‐NOx, VOC‐limited conditions where marginal VOC additions yield the highest incremental ozone formation (Carter 2012; Venecek et al. 2018). Thus, dryland VOC pulses may have different ozone‐forming potential depending on whether they occur under remote low‐NOx conditions or under the influence of transported urban, industrial, or traffic‐related NOx plumes. However, regional anthropogenic NO_x_ emissions frequently exceed the threshold required for high‐NOx (> 10 ppb) chemical regimes throughout Israel, indicating that these NOx levels could be sufficient for producing ozone and SOA from petrichor VOC emissions in Israel. Also, soil microbes could be a further significant NO_x_ emission source at rural locations, especially since these emissions strongly increase by rewetting (Yienger and Levy II 1995), similarly to petrichor VOC emissions. Finally, the concurrent emission pulse of VOCs and NO_x_ after rewetting in drylands could stimulate cloud condensation nuclei production (Makkonen et al. 2012), resulting in a coupling between biogenic emissions, aerosol, and precipitation.

Strong VOC emissions occurred across all climatic regions, although their magnitude and composition varied along the aridity gradient. Because drylands cover nearly half of Earth's terrestrial surface (Imminger et al. 2024; Reynolds et al. 2007) and are projected to expand under climate change, rainfall‐triggered microbial VOC pulses may represent an underappreciated episodic source of reactive carbon to the atmosphere. Changes in precipitation timing, drought duration, and rainfall intensity are likely to alter the frequency and magnitude of microbial activation events in dry soils, potentially changing episodic VOC emissions and their contribution to ozone and aerosol formation.

Despite their short duration, these episodic but spatially extensive pulses could be relevant for atmospheric processes. Similar to the well‐known Birch effect for CO_2_ pulses, rainfall‐driven microbial VOC emissions represent a rapid release of previously stored or newly synthesized carbon following drought, linking microbial physiology directly to atmospheric chemistry. Our findings suggest that rainfall‐driven microbial VOC emissions represent a source of reactive carbon from dryland soils. Because climate change is projected to intensify precipitation variability and expand drylands globally, the frequency and magnitude of these microbial emission events may increase in many regions. Current global VOC emission inventories and Earth system models largely focus on plant emissions and do not explicitly account for soil VOC emissions from drylands following rainfall events. Incorporating such microbial emissions may therefore improve global predictions of biosphere‐atmosphere interactions and atmospheric reactivity.

## 4 Conclusion

This study shows that rewetting dryland soils after prolonged drought triggers strong petrichor emissions that may be relevant for SOA and tropospheric O_3_ formation. These emissions included oxygenated VOCs, terpenoids, benzenoids, and sulfur‐containing compounds, and their composition varied systematically along the aridity gradient, consistent with contributions from both physicochemical release and renewed microbial activity. Upscaling indicated that such pulses may represent an underrecognized episodic source of reactive carbon to the atmosphere, with potential consequences for atmospheric oxidation capacity and aerosol formation. As drylands expand and precipitation regimes become more variable under climate change, rainfall‐triggered soil VOC emissions may become increasingly important for understanding biosphere‐atmosphere interactions.

## 5 Materials and Methods

### 5.1 Soil Collection

Soil cores were collected at the end of the dry season (end of August) along a ~160‐km in a north–south transect across the Judea Hills and Negev Desert of Israel, established to represent an aridity gradient (Bay et al. 2020, 2021) (Figure 1). Soils were not hydrated for at least 3 months. Collection sites were located within four distinct climatic regions, each distinguished by annual precipitation: sub‐humid (SH) shrubland (> 300 mm yr.^−1^), semi‐arid (SA) grassland (~200–300 mm yr.^−1^), arid (A) desert (~50–200 mm yr.^−1^), and hyper‐arid (HA) desert (< 50 mm yr.^−1^). Full data on soil moisture, temperature regimes, soil taxonomic order, soil properties and aridity index can be found in Supporting Information (Figures S1 and S2, Data S1, Tables S1 and S2). Five replicate soil cores were obtained from two sampling sites in each climatic region (“A” and “B,” distant ~2–10 km apart). Soil texture and soil water content (SWC) are provided in the supplements (Table S2).

To preserve soil structure and biocrust integrity, aluminum columns (56 mm × 40 mm; d × l) were inserted vertically into the soil until their upper edges were level with the biocrust surface. Cotton pads were placed on top of each column to protect the biocrust, and the column was carefully excavated and gently extracted from the soil. Then a second cotton pad was placed at the bottom, the pads were secured with aluminum foil, and the column was sealed with PET foils. The secured columns were transported to Helmholtz Munich (Germany) for analysis. Altogether, 40 columns were analyzed (5 replicates × 2 sites × 4 climatic regions). Samples were stored under controlled conditions (21°C ± 1°C; 50% ± 10% RH) until analysis.

### 5.2 Soil Rewetting

Soil columns were rewetted in two simulated rain events. Rewetting was achieved by slowly dropping 25 mL of Milli‐Q water in five sequential injections of 5 mL each at a rate of 1.5 mL min^−1^ through an injection port and using a glass syringe. This corresponds to a rainfall event of ~10 mm, which is a realistic single precipitation event in Israel, where annual rainfall ranges from > 600 mm in Mediterranean regions to < 50 mm in the hyper‐arid Negev Desert. The first rewetting followed > 3 months of drought, and the second after 31 days of desiccation (Figure S3). Five replicate soil cores were continuously monitored for 4 days after each rewetting event. Soils were incubated in 12 h light/dark cycles (135 ± 15 μmol m^−2^ s^−1^ PPFD) with temperatures of 28.4°C ± 0.2°C (day) and 22.8°C ± 0.1°C (night) and relative humidity of 11% ± 1% / 65% ± 2%, respectively (Figure S4).

### 5.3 VOC Collections

VOC fluxes were measured before and after the rewetting events under controlled environmental conditions using dynamic flow‐through cuvettes (Riedlmeier et al. 2017; Wenig et al. 2019). The original glass cuvettes were replaced by 10 1 L odorless PET chambers to enable simultaneous measurements during simulated rainfall. Purified air (200 mL min^−1^) continuously flushed each cuvette, maintaining VOC mixing ratios constant and below 10 ppbv, and CO_2_ at 420 ppmv (Figure S5). Outlet air was directed to a proton‐transfer‐reaction quadrupole mass spectrometer (PTR‐QMS) for online VOC analysis, a thermal desorption gas chromatography mass spectrometry (TD‐GC–MS) system for offline analysis, and an infrared gas analyzer for CO_2_ and H_2_O measurements. Cuvettes were switched automatically every 6 min, yielding a 1‐h temporal resolution. The first 4 min of each cycle were discarded to avoid carry‐over. The cuvettes were measured in randomized order. Full experimental details are provided in Method S2 and Figures S3–S6.

### 5.4 VOC Analyses by PTR‐QMS and TD‐GC–MS

Fluxes of VOCs were quantified online using proton‐transfer‐reaction quadrupole mass spectrometry (PTR‐QMS; Ionicon Analytik GmbH) and offline using thermal desorption gas chromatography–mass spectrometry (TD‐GC–MS; Gerstel GmbH/Agilent Technologies). Measurements were performed using established procedures (Ghirardo et al. 2012, 2020, 2016), and detailed information is provided in the Method S3. VOC fluxes were calculated from the difference between outlet and inlet mixing ratios, chamber flow rate, and soil surface area, and are reported as area‐based fluxes (Method S3).

A list of monitored protonated masses (PTR‐QMS) and chemical assignments is provided in Table S10. Assignments were based on TD‐GC–MS identification and authentic standards. VOCs collected on TD‐GC–MS adsorbent tubes were analyzed using both untargeted and targeted workflows. Untargeted feature detection was performed using MS‐DIAL (Tsugawa et al. 2015), whereas targeted identification and quantification were performed using Agilent GC–MS Enhanced ChemStation, version E.02.00.493 (Agilent Technologies), and using authentic reference standards, NIST/Wiley library matches, Kovats retention indices, and calibration curves. Detailed PTR‐QMS operating conditions, TD‐GC–MS parameters, calibration procedures, compound identification criteria, limits of detection, and flux calculations are provided in Method S3.

### 5.5 Gas Exchange (CO_2_ and H_2_O) Fluxes

CO_2_ and H_2_O fluxes were measured continuously using an infrared gas analyzer (IRGA; LI‐850, LI‐COR Biosciences). The instrument was calibrated with certified CO_2_ standard gases (Air Liquide Deutschland GmbH, Düsseldorf, Germany) covering a concentration range of 0–2000 ppmv. The H_2_O channel was calibrated using synthetic air humidified in a glass water bubbler and subsequently passed through a temperature‐controlled dew‐point/cold‐trap system (Walz, Germany), which set the gas stream to a defined dew point and known water‐vapor content before entering the analyzer.

CO_2_ and H_2_O fluxes were calculated from the difference between outlet and inlet concentrations, chamber flow rate, and the enclosed soil surface area, following (Ghirardo et al. 2011). Fluxes were normalized to soil surface area and calculated separately for each mesocosm, climatic region, and sampling phase. Positive CO_2_ and H_2_O fluxes indicate net release from the soil to the chamber atmosphere, whereas negative values of CO_2_ indicate net uptake.

### 5.6 Analysis of Bacterial Community

Bacterial community profiles across the aridity gradient were obtained from a previous dry‐season soil sampling campaign conducted in the same climatic regions and long‐term study sites (Bay et al. 2021). These samples were not the same soil cores used for VOC emission measurements, and therefore, the community data were used to provide the site‐level microbial context. We re‐analyzed the 16S rRNA amplicon dataset (Bay et al. 2021). Soil samples were collected from the same locations and separated into biocrust (0–2 cm) and topsoil (2–10 cm). DNA was extracted and 16S rRNA gene amplicon sequencing was performed as previously described (Bay et al. 2021). We re‐processed the sequence reads to generate updated community composition profiles for the present study.

### 5.7 Assessment of Potential Impact on SOA and O_3_

The petrichor molar emission rates (ER) (pmol m^−2^ s^−1^) were converted to g m^−2^ h^−1^ using the molecular weight (MW) of each compound. Estimates of the maximum incremental reactivity (MIR) and SOA yields (Y_SOA) for each petrichor compound (Gu et al. 2021) were used to estimate OFP and SOAP for each compound as:

OFP=ER×MIR

SOAP=ER×Y_SOA

### 5.8 Statistical Analysis

The VOC fluxes were analyzed by multivariate data analysis, *t*‐tests, and ANOVA to evaluate how soil VOC emission profiles differed among rewetting stages and climatic regions along the aridity gradient. Principal component analysis (PCA) and orthogonal partial least squares regression (OPLS) were performed using SIMCA‐P v.13.0.3.0 (Umetrics, Umeå, Sweden), essentially as before (Ghirardo et al. 2016; Riedlmeier et al. 2017; Weikl et al. 2016) and reported below.

For multivariate analysis, data were log_10_‐transformed, mean‐centered, and Pareto‐scaled before multivariate modeling. Principal component analysis (PCA) was first performed as an unsupervised quality‐control step to inspect overall sample structure and identify potential outliers. Orthogonal partial least squares (OPLS) models were then used to identify VOCs associated with soil rewetting stage and aridity. For analyses of rewetting effects, VOC emission rates were used as independent variables (X), whereas rewetting stage was used as the dependent variable (Y), with dry soil, first rewetting, and second rewetting treated as categorical classes. Additional OPLS models were constructed separately for the first and second rewetting events to evaluate differences among the climatic regions. To assess the relationship between VOC fluxes and the aridity gradient, climatic regions were also encoded as an ordered Y‐variable (1–4) representing increasing aridity. Rewetting stage was treated as a categorical factor with three levels: dry soil, first rewetting, and second rewetting. Score plots show similarities among samples based on their VOC emission profiles, with samples plotting closer together having more similar VOC composition and fluxes. Loading or coefficient plots indicate which VOCs contributed most strongly to the separation among stages or climatic regions. Positive or negative coefficients indicate the direction of association with stage‐ or region‐associated VOC responses. OPLS score plots were used to visualize differences in VOC emission profiles among samples, rewetting stages, and climatic regions. Loading plots and model coefficients were used to identify VOCs contributing most strongly to group separation or to the aridity‐related gradient. Model performance was assessed using R^2^X(cum), R^2^Y(cum), Q^2^Y(cum), the root mean square error of estimation (RMSEE), and the root mean square error of cross‐validation (RMSEcv). Model significance was tested using cross‐validated analysis of variance (CV‐ANOVA) (Eriksson et al. 2008) using a 99% confidence level on parameters and seven cross‐validation groups, with *p* < 0.05 considered significant.

Hierarchical clustering based on Spearman correlations, Kruskal–Wallis tests, PERMANOVA, and multiple‐testing corrections (Benjamini–Hochberg procedure) were performed in R (R Core Team 2021). Differences in bacterial community composition across habitat types and climatic regions were assessed using permutation‐based multivariate analyses on Hellinger‐transformed 16S rRNA amplicon data, with tests for both centroid shifts and dispersion effects (see Method S4 for details).

## Author Contributions

**Andrea Ghirardo:** conceptualization, methodology, investigation, writing – original draft, funding acquisition, validation, visualization, writing – review and editing, data curation, resources. **Benjamin W. Poodiack:** methodology, investigation, visualization, data curation. **Hagar Siebner:** conceptualization, methodology, investigation, writing – review and editing. **Matan K. Jaffe:** methodology, writing – review and editing. **Baris Weber:** methodology, investigation. **Jörg‐Peter Schnitzler:** writing – review and editing, resources. **Michael Bonkowski:** writing – review and editing, funding acquisition. **Osnat Gillor:** conceptualization, investigation, writing – original draft, funding acquisition, validation, visualization. **Alex B. Guenther:** investigation, writing – review and editing, visualization, methodology, data curation.

## Funding

Deutsche Forschungsgemeinschaft (DFG), grant 516726624 (AG, MB, OG).

National Science Foundation and Binational Science Foundation (NSF‐BSF) grant 8100103 (OG, HS, BP).

## Conflicts of Interest

The authors declare no conflicts of interest.

## Supporting information


**Figure S1:** Soil characteristics.
**Figure S2:** Soil physicochemical properties.
**Figure S3:** Experimental timeline.
**Figure S4:** Air temperature.
**Figure S5:** Schematic of the dynamic flow‐through cuvette enclosure system.
**Figure S6:** Soil cores.
**Figure S7:** Volatiles strongly correlating with rewetting.
**Figure S8:** Geosmin.
**Figure S9:** Correlation coefficients of VOCs to climatic regions.
**Figure S10:** VOC emission rates of the volatiles correlated with the climate regions.
**Figure S11:** Emission dynamics of oxygenated VOCs.
**Figure S12:** Emission dynamics of terpene‐related VOCs.
**Figure S13:** Emission dynamics of sulfur‐containing and other VOCs.
**Figure S14:** Correlation heatmap of PTR‐QMS ion signals.
**Figure S15:** Correlation heatmap of VOC emission rates and CO_2_ and H_2_O fluxes.
**Figure S16:** Petrichor‐driven VOC mass, O_3_ and SOA formation potential.
**Table S2:** Soil texture and water content analyses.
**Table S3:** Soil bacterial community differences.
**Table S4:** Homogeneity of dispersion.
**Table S5:** Climatic region effect on bacteria composition.
**Table S6:** Soil type effect on bacteria composition.
**Table S9:** List of m/z measured with PTR‐QMS.
**Method S1**. Soil analysis.
**Method S2**. Experimental design.
**Method S3**. VOC analysis.
**Method S4**. Statistical analysis of bacterial diversity and community composition.

**Table S1:** Microbial compositions.

**Table S7:** ID of VOCs.

**Table S8:** VOC emission rates.

**Data S1:** Supporting Information.

## Data Availability

All data are available in the Supporting Information or on OSF (https://doi.org/10.17605/OSF.IO/JC65S), and on the Sequence Read Archive under BioProject accession number PRJNA657906.
